# Plasma proteomics improves prediction of coronary plaque progression

**DOI:** 10.1093/ehjci/jeae313

**Published:** 2024-12-10

**Authors:** Jordan M Kraaijenhof, Nick S Nurmohamed, Michiel J Bom, E L Gaillard, Shirin Ibrahim, Cheyenne Y Y Beverloo, R Nils Planken, G Kees Hovingh, Ibrahim Danad, Erik S G Stroes, Paul Knaapen

**Affiliations:** Department of Vascular Medicine, Amsterdam University Medical Centers, University of Amsterdam, Amsterdam, The Netherlands; Department of Vascular Medicine, Amsterdam University Medical Centers, University of Amsterdam, Amsterdam, The Netherlands; Department of Cardiology, Amsterdam University Medical Centers, Vrije Universiteit Amsterdam, Amsterdam, The Netherlands; Department of Cardiology, Amsterdam University Medical Centers, Vrije Universiteit Amsterdam, Amsterdam, The Netherlands; Department of Vascular Medicine, Amsterdam University Medical Centers, University of Amsterdam, Amsterdam, The Netherlands; Department of Cardiology, Amsterdam University Medical Centers, Vrije Universiteit Amsterdam, Amsterdam, The Netherlands; Department of Vascular Medicine, Amsterdam University Medical Centers, University of Amsterdam, Amsterdam, The Netherlands; Department of Vascular Medicine, Amsterdam University Medical Centers, University of Amsterdam, Amsterdam, The Netherlands; Department of Radiology and Nuclear Medicine, Amsterdam University Medical Centers, University of Amsterdam, Amsterdam, The Netherlands; Department of Vascular Medicine, Amsterdam University Medical Centers, University of Amsterdam, Amsterdam, The Netherlands; Department of Cardiology, Radboud University Medical Center, Radboud University, Nijmegen, The Netherlands; Department of Vascular Medicine, Amsterdam University Medical Centers, University of Amsterdam, Amsterdam, The Netherlands; Department of Cardiology, Amsterdam University Medical Centers, Vrije Universiteit Amsterdam, Amsterdam, The Netherlands

**Keywords:** coronary computed tomography angiography, plasma proteomics, plaque progression, atherosclerotic cardiovascular disease

## Abstract

**Aims:**

Coronary computed tomography angiography (CCTA) offers detailed imaging of plaque burden and composition, with plaque progression being a key determinant of future cardiovascular events. As repeated CCTA scans are burdensome and costly, there is a need for non-invasive identification of plaque progression. This study evaluated whether combining proteomics with traditional risk factors can detect patients at risk for accelerated plaque progression.

**Methods and results:**

This long-term follow-up study included 97 participants who underwent two CCTA scans and plasma proteomics analysis using the Olink platform. Accelerated plaque progression was defined as rates above the median for percent atheroma volume (PAV), percent non-calcified plaque volume (NCPV), and percent calcified plaque volume (CPV). High-risk plaque (HRP) was identified by positive remodelling or low-density plaque at baseline and/or follow-up. Significant proteins associated with PAV, NCPV, CPV, and HRP development were incorporated into predictive models. The mean baseline age was 58.0 ± 7.4 years, with 63 (65%) male, and a median follow-up of 8.5 ± 0.6 years. The area under the curve (AUC) for accelerated PAV progression increased from 0.830 with traditional risk factors and baseline plaque volume to 0.909 with the protein panel (*P* = 0.023). For NCPV progression, AUC improved from 0.685 to 0.825 (*P* = 0.008), while no improvement was observed for CPV progression. For HRP development, AUC increased from 0.791 to 0.860 with the protein panel (*P* = 0.036).

**Conclusion:**

Integrating proteomics with traditional risk factors enhances the prediction of accelerated plaque progression and high-risk plaque development, potentially improving risk stratification and treatment decisions without the need for repeated CCTAs.

## Graphical Abstract

**Graphical Abstract:**
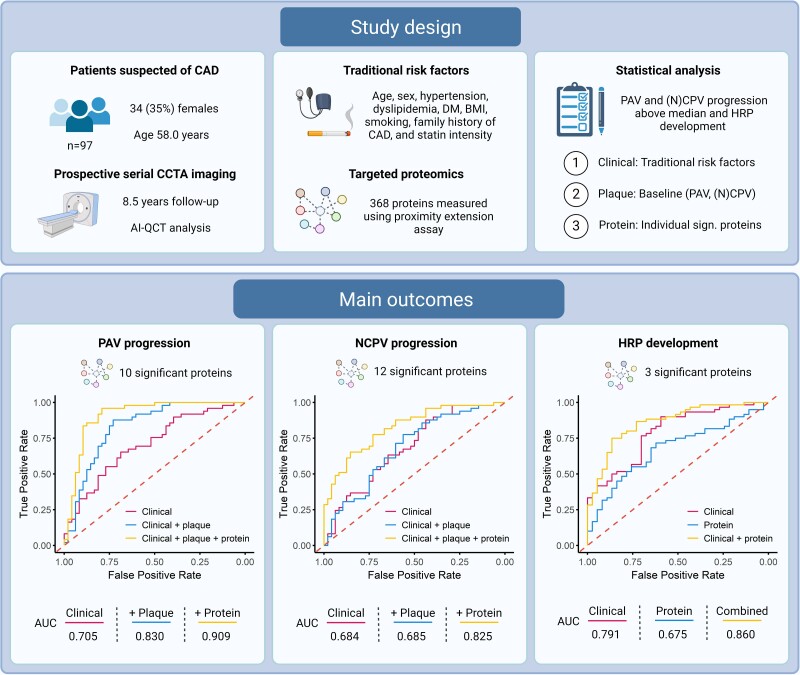
A total of 97 participants who underwent serial CCTA and proteomics analysis were included. Accelerated plaque progression was defined as rates above the median for PAV, NCPV, and CPV. HRP was identified by the presence of positive remodelling or low-density plaque at baseline and/or follow-up. For accelerated PAV, NCPV, and CPV progression, a clinical model incorporating traditional risk factors was established. Baseline plaque volumes for respectively PAV, NCPV, and CPV were then integrated into this model to create the clinical + baseline plaque model. Significant proteins associated with accelerated PAV, NCPV, CPV progression were identified and added, resulting in the clinical + baseline plaque + protein model. This model demonstrated superiority for PAV and NCPV compared with the clinical and clinical + baseline models. For HRP, models were constructed using clinical traditional risk factors, significant proteins, and a combination of both (clinical + protein). The combined clinical + protein model showed superiority over the clinical model alone. Results for CPV models are not shown. CAD, coronary artery disease; CCTA, coronary computed tomography angiography; AI-QCT, artificial intelligence-based quantitative CT image analysis software; DM, diabetes mellitus; BMI, body mass index; PAV, percent atheroma volume; NCPV, percent non-calcified plaque volume; CPV, percent calcified plaque volume; HRP, high-risk plaque; AUC, area under the curve; CI, confidence interval. Created with BioRender.com.

## Introduction

Determining the risk for atherosclerotic cardiovascular disease (ASCVD) is crucial for optimizing treatment to prevent cardiovascular events.^1,2^ Recent advances in imaging have been shown to improve risk estimation over traditional risk factors.^3–6^ In particular, the development of coronary computed tomography angiography (CCTA) derived plaque burden and high-risk plaque (HRP) characteristics were found to be associated with an increased risk of future cardiovascular events.^5–7^ Studies where serial CCTAs were performed showed that non-obstructive lesions typically enlarge swiftly before the onset of an acute cardiovascular event, whereas lesions that remain stable over time rarely lead to such events.^8,9^ However, the rate of plaque progression varies significantly between patients,^10^ making it essential to identify those with progressive plaques. Given that frequent CCTA scans are costly as well as burdensome due to radiation exposure, plasma markers hold the promise to provide an effective approach to identify those patients at higher risk of accelerated plaque progression.

The plasma proteome has been put forward as a potential method providing detailed insights into the mechanisms driving cardiovascular disease and acting as a predictive marker for disease prognosis, which may potentially provide guidance for treatment decision-making.^11^ We previously reported that targeted protein panels assisted in identifying high-risk coronary plaques in patients with suspected coronary artery disease (CAD).^12^ The application of plasma proteomics also improved the precision of cardiovascular risk prediction across both primary and secondary prevention cohorts.^13–17^ It remains to be established if targeted proteomics also helps to identify subjects at highest risk for increased plaque progression and/or development of HRP features.

In the present study, we set out to elucidate whether plasma proteomics increased the prediction of plaque progression over and above traditional cardiovascular risk factors. To this end, we assessed the predictive value for accelerated CCTA plaque progression and HRP development in high-risk patients comparing a proteomics panel with traditional risk factors during a long-term follow-up period.

## Methods

### Study population

The present investigation is a sub-study of the PACIFIC-1 (Prospective Comparison of Cardiac PET/CT, SPECT/CT perfusion imaging and CT coronary angiography with invasive coronary angiography) trial.^18^ The cohort included 208 patients, presenting with new onset, stable chest pain with a suspicion of CAD undergoing CCTA imaging. Participants were eligible if they were aged 40 years or older and had an intermediate likelihood of CAD based on the Diamond and Forrester criteria. Exclusion criteria encompassed renal insufficiency (eGFR <45 mL/min), a documented history of chronic obstructive pulmonary disease or chronic asthma, any previous diagnosis of CAD, atrial fibrillation, or second or third degree atrioventricular block. An invitation for second CCTA imaging was extended to all participants, irrespective of their symptomatology or medical history.^19^ The study was approved by the local ethics committee and conducted in accordance with the Declaration of Helsinki. All participants provided written informed consent. From the initial cohort of 208 participants, 103 underwent a second CCTA imaging study, of whom a total of 97 individuals had proteomics data available at baseline.

### Proteomics analysis

Details on proteomic analysis have been described previously.^12^ Briefly, the analysis was performed at baseline CCTA imaging utilizing proximity extension assays from Olink Biosciences (Uppsala, Sweden). The study employed 368 proteins in the Cardiovascular II, Cardiovascular III, Cardiometabolic, and Inflammation panels. The assay's output is reported in Normalized Protein eXpression (NPX) units, a semi-quantitative measure on a log2 scale, with higher NPX values indicating greater protein expression levels. Samples not meeting quality control standards were omitted from the study (*n* = 6). For proteins with fewer than 20% of measurements below this threshold, the missing values were replaced by substituting the limit of detection divided by two.^12^ For this study, we utilized a panel of 50 proteins identified by a machine learning model from a pool of 368 proteins in a previous study of primary prevention patients.^15^ This selection demonstrated superior performance over the conventional clinical risk model in both the derivation and validation cohorts.

### CCTA imaging and analysis

All participants initially underwent both coronary artery calcium (CAC) scoring and CCTA imaging with a 256-slice scanner from Philips Healthcare, Best, the Netherlands, as previously detailed.^20,21^ During the follow-up phase, CAC scoring and CCTA were performed using a third-generation dual-source CT scanner (SOMATOM Force, Siemens Healthineers, Germany). CCTA images were evaluated using an artificial intelligence-based software (Atherosclerosis Imaging Quantitative Computed Tomography, AI-QCT; Cleerly Inc., Denver, CO, USA).^22^ To mitigate variations in coronary artery volume, coronary plaque volume was normalized against vessel volume, expressed as plaque volume divided by vessel volume times 100%. These normalized numbers were presented as percent atheroma volume (PAV), percent non-calcified plaque volume (NCPV) and percent calcified plaque volume (CPV). Additionally, arterial remodelling was assessed by comparing lesion diameter to the normal reference diameter. In the sequential analysis, any vessel compromised by motion artefacts, poor opacification, beam hardening, or other artefacts was excluded from both baseline and follow-up imaging analyses, where scanner specific thresholds were used. Patients who received stents between the two CCTA scans were excluded to ensure consistency in comparisons. In addition to plaque volumes, the study examined the occurrence of HRP features. A HRP was defined as having positive remodelling and/low-density plaque. If these features were observed at either the baseline or follow-up, it was considered the development of HRP.

### Study outcomes

The primary outcome was defined as PAV progression during the follow-up period. Secondary outcomes were the progression of NCPV, CPV, and development of HRP.

### Statistical analysis

First, the association between baseline proteomic expression and the progression of PAV, NCPV, and CPV was evaluated using a linear regression model, adjusted for age, sex, and clinical risk factors including history of hypertension, history of hypercholesterolemia (prior or baseline total cholesterol ≥6.5 mmol/L), diabetes mellitus type 2, body mass index, smoking status, family history of CAD, and statin intensity. The Benjamini–Hochberg method was utilized to account for multiple testing. Protein–protein interaction networks were analysed and depicted using STRING v11 (string-db.org).

To assess the predictive capability of the proteomic panel on PAV, NCPV, and CPV progression, three distinct models were developed: a clinical model, a clinical + baseline plaque model, and clinical + baseline plaque + protein model. The clinical model incorporated age, sex, and clinical risk factors hypertension, hypercholesterolemia, diabetes mellitus type 2, body mass index, smoking status, family history of CAD, and statin intensity. The clinical + baseline plaque model included the baseline plaque volume of the respective plaque type. The clinical + baseline plaque + protein model additionally incorporated those proteins that were significant prior to the correction for multiple testing with a maximum of 10 proteins. For each model, a receiver operating characteristic (ROC) area under the curve (AUC) was calculated to evaluate the prediction accuracy for the progression above the median. The predictive capability of the proteomic panel on HRP development, either at baseline and/or at follow-up, was evaluated in a clinical model, a protein model and in a clinical + protein model. The DeLong test was used to calculate comparisons between the models’ predictive performances.^23^ To address potential overfitting, Bootstrap validation was conducted in accordance with the TRIPOD statement guidelines.^24^ Specifically, bootstrap samples were generated by sampling with replacement from the original dataset, with a new model developed for each sample to assess its performance. The discrepancy between the model's performance on the bootstrap and original datasets was used to calculate optimism. This process was repeated 1000 times, with the mean optimism used to adjust the initial performance estimates.

Network analyses were conducted on significant proteins using the STRING database (https://string-db.org, accessed on 5 June 2024), employing the full-string network mode to capture both physical and functional protein–protein interactions.^25^ This enabled a comprehensive assessment of network connectivity and was integral to subsequent Gene Ontology (GO) enrichment analysis, which identified biological processes significantly associated with the proteins in plaque progression.

Data are presented as mean ± standard deviation (SD) for normally distributed variables, and median with inter-quartile range [IQR] for variables that are not. For categorical variables, frequencies and percentages are provided. Where applicable, the independent sample *t*-tests and Mann–Whitney *U* tests were employed. Clinical and imaging-based parameters were associated with PAV progression in both univariate and age- and sex-adjusted linear regression models. Two-sided *P*-values of 0.05 or lower were considered statistically significant. All statistical analyses were conducted using RStudio software, version 4.3.2 (R Foundation, Vienna, Austria).

## Results

### Baseline patient and imaging characteristics

The mean age of the 97 participants was 58.0 ± 7.4 years at baseline, and 63 individuals (65%) were male (*Table 1*). Twenty (20.6%) participants had diabetes, and 42 (43.3%) had hypertension. The follow-up imaging was conducted on average 8.5 ± 0.6 years after the initial assessment. At baseline, the PAV was 2.94% [IQR: 0.74, 8.16] (see Supplementary data online, *Table S1*), the NCPV 2.10% [IQR: 0.59, 4.95] and the NCP 0.85% [0.00, 2.28]. HRPs were present in 43 (44.3%) patients at baseline and in 47 (48.5%) patients at follow-up. A total of 60 (61.9%) patients had either a HRP at baseline and/or follow-up. The progression rates for PAV, NCPV, and CPV were 1.17% [IQR: 0.29, 4.08], 0.45% [IQR: −0.07, 2.27], and 0.58% [IQR: 0.21, 2.05], respectively (see Supplementary data online, *Table S1*). Type 2 diabetes mellitus was significantly associated with PAV progression. In the age- and sex-adjusted model, diabetes mellitus showed a beta coefficient of 1.914 (*P* = 0.034) (see Supplementary data online, *Table S2*). Baseline PAV and HRP were also significantly associated with PAV progression, with beta coefficients of 0.112 (*P* = 0.023) and 1.692 (*P* = 0.016), respectively, in the age- and sex- adjusted model. Stratification by median PAV progression showed that those above the median were older (59.8 vs. 56.1 years, *P* = 0.012) (*Table 1*).

**Table 1:** Baseline characteristics

Characteristics	Total cohort (*n* = 97)	PAV progression < median (*n* = 48)	PAV progression ≥ median (*n* = 49)	*P*-value
Follow-up (years)	8.5 (0.6)	8.5 (0.6)	8.6 (0.7)	0.738
Age (years)	58.0 (7.4)	56.1 (6.6)	59.8 (7.8)	**0**.**012**
Male sex (%)	63 (64.9)	29 (60.4)	34 (69.4)	0.476
Current smoker (%)	20 (20.6)	11 (22.9)	9 (18.4)	0.762
Diabetes mellitus (%)	15 (15.5)	4 (8.3)	11 (22.4)	0.101
Hypertension (%)	42 (43.3)	17 (35.4)	25 (51.0)	0.178
Hypercholesterolemia (%)	38 (39.2)	16 (33.3)	22 (44.9)	0.338
Family history of CAD (%)	53 (54.6)	28 (58.3)	25 (51.0)	0.604
BMI (kg/m^2^)	26.7 (3.8)	26.7 (3.9)	26.7 (3.8)	0.990
Statin intensity (%)				
No statin	25 (25.8)	15 (31.2)	10 (20.4)	0.371
Low-intensity	1 (1.0)	0 (0.0)	1 (2.0)	
Medium-intensity	57 (58.8)	25 (52.1)	32 (65.3)	
High-intensity	14 (14.4)	8 (16.7)	6 (12.2)	
Beta blockers use (%)	59 (60.8)	28 (58.3)	31 (63.3)	0.772
Aspirin use (%)	83 (85.6)	39 (81.2)	44 (89.8)	0.364
Calcium blockers use (%)	26 (26.8)	12 (25.0)	14 (28.6)	0.867
Total cholesterol (mmol/L)	4.5 (1.0)	4.5 (1.2)	4.5 (0.9)	0.874
LDL cholesterol (mmol/L)	2.5 (1.0)	2.5 (1.1)	2.5 (0.9)	0.742
HDL cholesterol (mmol/L)	1.4 (0.5)	1.4 (0.5)	1.4 (0.6)	0.834
Triglycerides (mmol/L)	1.2 [0.9, 1.6]	1.1 [0.9, 1.6]	1.4 [0.9, 1.7]	0.362
Lipoprotein(a) (nmol/L)	22 [10, 113]	22 [10, 60]	24 [1, 138]	0.478
eGFR (mL/min/1.73 m2)	100.3 (21.1)	100.7 (22.6)	100.0 (19.9)	0.859

Baseline characteristics per remnant-cholesterol quartile. Continuous variables with a normal distribution are reported as mean (SD). Continuous variables with a non-normal distribution are reported as median ± IQR. ANOVA testing was performed for normally distributed continuous variables, while Kruskal–Wallis testing was used for non-normally distributed variables. BMI, body mass index; LDL, low-density lipoprotein; HDL, high-density lipoprotein; eGFR, estimated glomerular filtration rate.

### Proteomic panel predicts PAV and NCPV progression as well as HRP development

**Figure 1:**
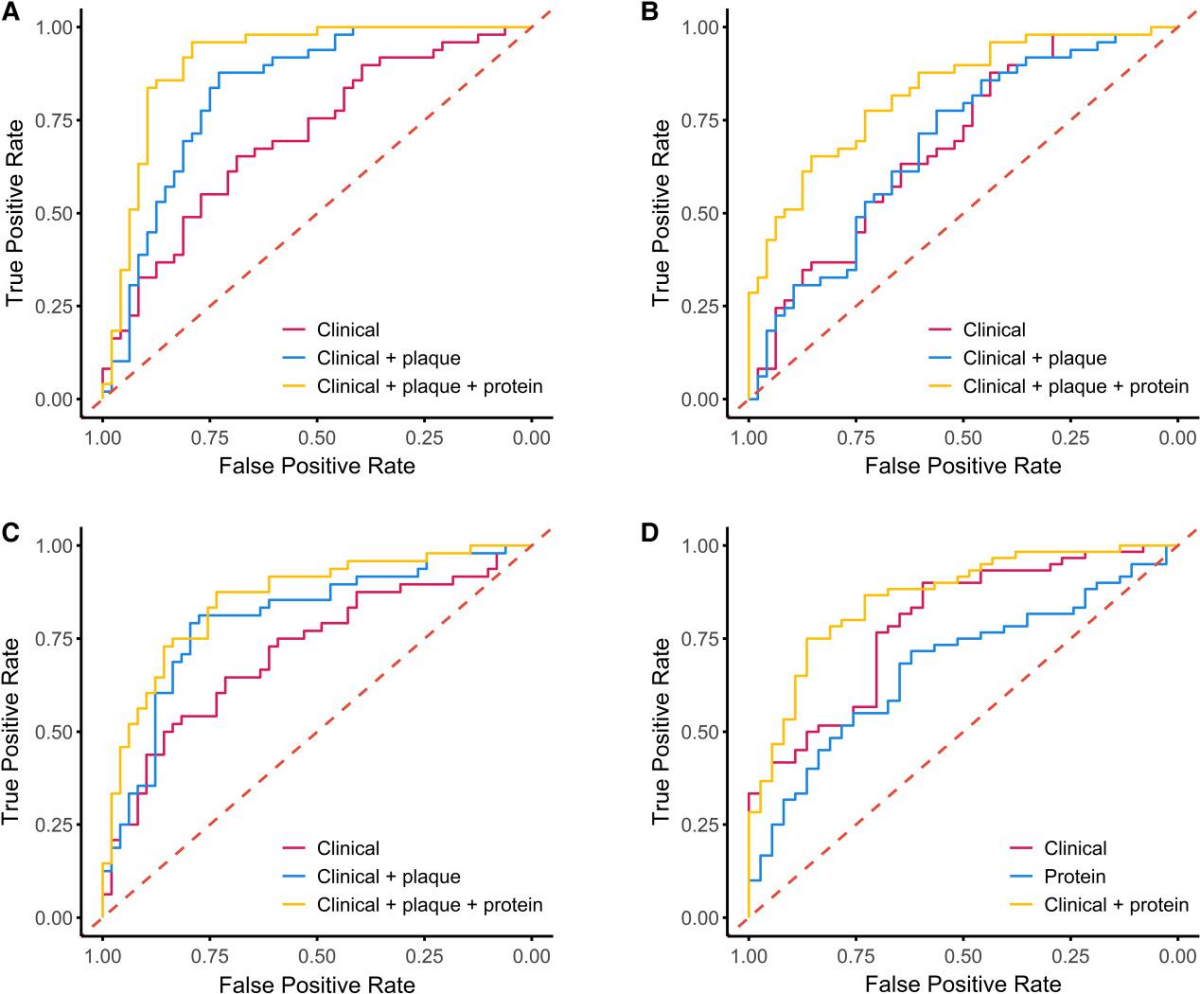
ROC curves of CCTA plaque volume progression. ROC curve of the statistical models for (*A*) percent atheroma volume, (*B*) percent NCPV, (*C*) percent CPV progression, and (*D*) HRP development. ROC, receiver operating characteristics.

In the multivariable adjusted linear regression analysis, 10, 12, and 3 proteins were significantly associated with PAV, NCPV, and CPV plaque progression respectively (*Table 2*). Three proteins were significantly associated with HRP development. The prediction of PAV progression using the clinical model, which included traditional cardiovascular risk factors resulted in an AUC of 0.705 (95% confidence interval (CI): 0.602–0.801) (*Table 3*; *Figure 1A*). Addition of baseline plaque improved the AUC significantly to 0.830 (95% CI: 0.745–0.915); a 0.095 (95% CI: 0.026–0.207, *P* = 0.003) increase. Subsequent inclusion of the protein panel resulted in an AUC of 0.909 (95% CI: 0.844–0.974); a significant improvement, calculated by bootstrapping, over the clinical and baseline plaque model of 0.079 (95% CI: 0.012–0.115, *P* = 0.023). The optimism corrected AUCs for PAV, NPCV, and CPV were 0.650 (0.579–0.733), 0.816 (0.730–0.912), and 0.871 (0.818–0.942), respectively (*Table 3*). For NCPV, the clinical model had an AUC of 0.684 (95% CI: 0.588–0.790) (*Table 3*; *Figure 1B*). Addition of baseline plaque demonstrated a similar AUC of 0.685 (95% CI: 0.579–0.792), ΔAUC 0.008 (−0.012–0.050, *P* = 0.928). After inclusion of the proteins, the model showed an AUC of 0.825 (95% CI: 0.744–0.906); a significant improvement of the AUC by 0.152 (95% CI: 0.075–0.244, *P* = 0.008) compared with the clinical and baseline plaque model. In the analysis for CPV, the clinical model led to an AUC of 0.716 (95% CI: 0.613–0.820) (*Table 3*; *Figure 1C*). Inclusion of baseline plaque resulted in an AUC of 0.803 (95% CI: 0.713–0.893), an improvement of 0.084 (95% CI: 0.011–0.164, *P* = 0.023). Incorporation of the protein panel resulted in an AUC of 0.855 (95% CI: 0.780–0.931), which was significantly higher compared with the clinical model, ΔAUC 0.137 (0.055–0.232, *P* = 0.004), but not compared with the clinical and baseline plaque model.

**Table 2:** Significant proteins associated with plaque progression

	Coefficient	Intercept	*P*-value
A. Percent atheroma volume progression			
GDF-15	0.076	1.833	0.0001*
CCL4	0.084	5.630	0.0005*
CCL3	0.038	1.188	0.012
OPG	0.034	3.256	0.021
TRAIL-R2	0.025	4.352	0.024
U-PAR	0.036	3.617	0.035
SLAMF1	0.032	−0.001	0.036
TNF-R1	0.037	4.536	0.036
ADM	0.026	4.937	0.043
IL-6	0.050	0.343	0.048
B. Percent NCPV progression			
GDF-15	0.114	1.840	0.0001*
CCL4	0.110	5.627	0.003
CCL3	0.061	1.194	0.008
IL-6	0.101	0.364	0.008
TRAIL-R2	0.042	4.357	0.014
TFF3	0.060	5.132	0.016
U-PAR	0.061	3.624	0.017
KIM1	0.080	3.078	0.027
TGF-α	0.050	2.136	0.031
OPG	0.047	3.256	0.035
TNF-R1	0.054	4.538	0.044
ADM	0.039	4.938	0.049
C. Percent CPV progression			
CCL4	0.141	5.461	0.002
GDF-15	0.111	1.692	0.003
CCL3	0.058	1.116	0.037
D. HRP development			
CCL3	0.258	1.194	0.017
SCF	−0.181	8.389	0.033
MMP-12	0.272	4.773	0.050

Significant proteins associated with (A) percent atheroma volume, (B) percent NCPV, (C) percent CPV progression, and (D) HRP development over 10-year follow-up, corrected for age, sex, BMI, hypertension, hypercholesterolemia, smoking, and family history of CAD. Asterisks represent significance after Benjamini–Hochberg correction. ADM, adrenomedullin; CCL3, chemokine ligand 3; CCL4, chemokine ligand 4; GDF-15, growth differentiation factor 15; IL-6, interleukin 6; IL-18BP, interleukin-18 binding protein; OPG, osteoprotegerin; OPN, osteopontin; SELL, selectin L; SLAMF1, sphingosine-1-phosphate receptor 1; TFF3, trefoil factor 3; TGF-alpha, transforming growth factor alpha; TNF-R1, tumour necrosis factor receptor 1; TNF-R2, tumour necrosis factor receptor 2; TRAIL-R2, TNF-related apoptosis-inducing ligand receptor 2; U-PAR, urokinase plasminogen activator surface receptor; VEGFA, vascular endothelial growth factor A; CAD, coronary artery disease; BMI, body mass index.

**Table 3:** AUC performance metrics

	Clinical model	Clinical + baseline plaque model	Clinical + baseline plaque + protein model
Percent atheroma plaque volume progression			
AUC (95% CI)	0.705 (0.602–0.801)	0.830 (0.745–0.915)	0.909 (0.844–0.974)
AUC (95% CI) corrected	0.650 (0.579–0.733)	0.816 (0.730–0.912)	0.871 (0.818–0.942)
*P*-value	Ref	**0.003**	**<0.001**
		Ref	**0.023**
Percent NCPV progression			
AUC (95% CI)	0.684 (0.588–0.790)	0.685 (0.579–0.792)	0.825 (0.744–0.906)
AUC (95% CI) corrected	0.650 (0.579–0.733)	0.620 (0.545–0.715)	0.749 (0.681–0.825)
*P*-value	Ref	0.928	**0.008**
		Ref	**0.008**
Percent CPV progression			
AUC (95% CI)	0.716 (0.613–0.820)	0.803 (0.713–0.893)	0.855 (0.780–0.931)
AUC (95% CI) corrected	0.671 (0.578–0.770)	0.771 (0.680–0.871)	0.811 (0.757–0.912)
*P*-value	Ref	**0.023**	**0.004**
		Ref	0.101
	Clinical model	Protein model	Combined model
HRP development			
AUC (95% CI)	0.791 (0.700–0.882)	0.675 (0.567–0.783)	0.860 (0.785–0.935)
AUC (95% CI) corrected	0.750 (0.677–0.838)	0.652 (0.563–0.746)	0.820 (0.765–0.888)
*P*-value	Ref	0.109	**0.036**

Significant *P*-values (<0.05) are presented in bold. AUC for the clinical model, protein model, and combined model for percent atheroma volume and percent NCPV, percent CPV, and HRP development predicting plaque progression above the median. AUC correction is calculated. AUC, area under the curve; CI, confidence interval.

**Figure 2:**
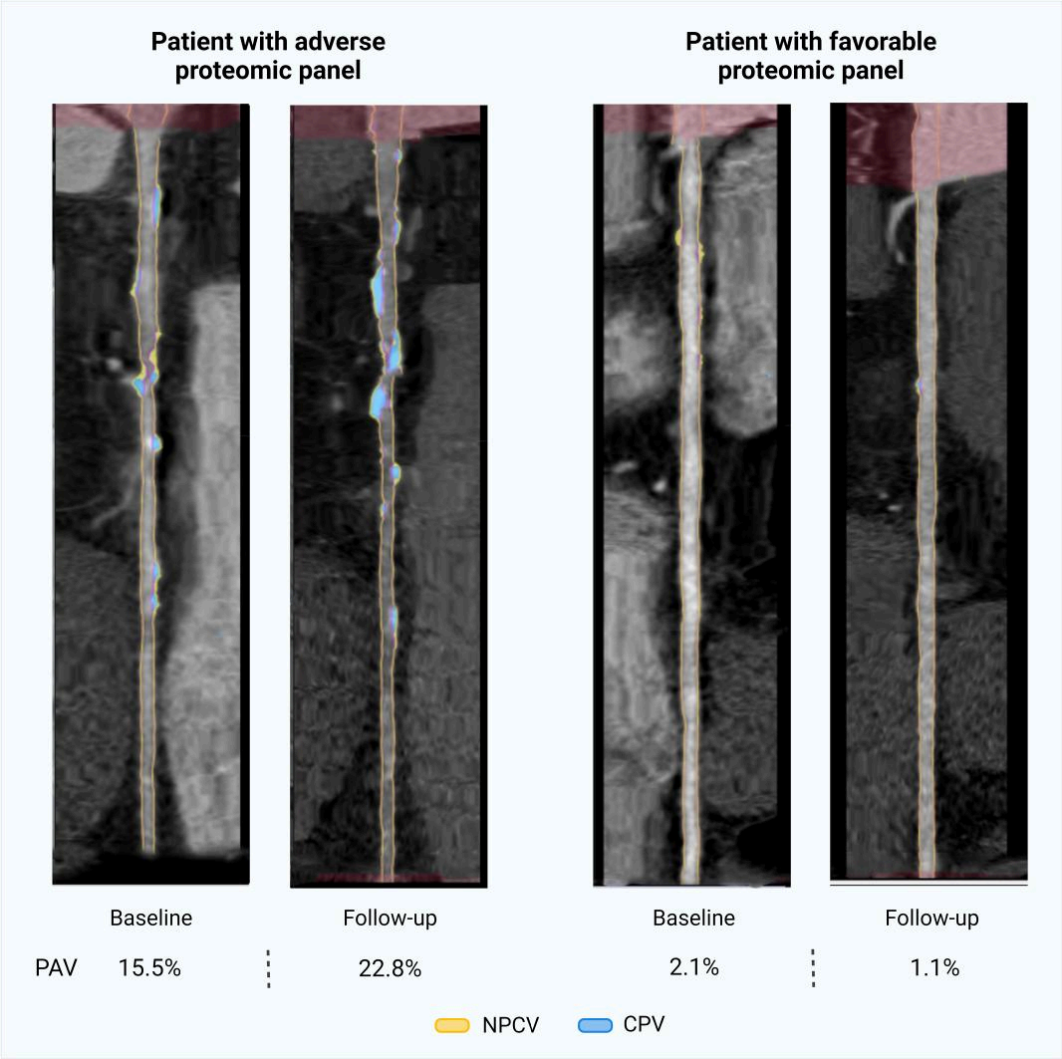
Visual comparison of PAV plaque progression. Example of AI-QCT analysis of the RCA in a patient with adverse (left) and favourable (right) inflammatory proteomic panels. PAV, percent atheroma volume; NCPV, percent non-calcified percent volume; CPV, percent calcified volume; AI-QCT, artificial intelligence–guided quantitative coronary computed tomography angiography analysis; RCA, right coronary artery. Created with BioRender.com.

In the HRP development model, the clinical model resulted in an AUC of 0.791 (95% CI: 0.700–0.882) (*Table 3; Figure 1D*) whereas the protein model had an AUC of 0.675 (95% CI: 0.567–0.783). The combined model’s AUC was 0.860 (95% CI: 0.785–0.935); a significant improvement by 0.062 (95% CI: 0.011–0.142, *P* = 0.036) compared with the clinical model (*Table 3*). The average optimism in the plaque progression models ranged from 0.041 to 0.055 in the clinical models, 0.014 to 0.065 in the in the clinical and baseline plaque models and 0.038 to 0.076 in the clinical plus baseline plaque plus protein models. The average optimism in the HRP development models were 0.041 in the baseline model, 0.024 in the protein model, and 0.041 in the combined model. A visual representation of the PAV plaque progression AI-QCT analysis is presented in *Figure 2*.

### Pathway analysis of associated proteins

There was an overlap in individual proteins associated with accelerated plaque progression across the different plaque composition measures (*Table 2*). Chemokine Ligand 3 (CCL3) emerged as pivotal across all types of accelerated plaque progression in addition to HRP development, whereas Growth Differentiation Factor 15 (GDF-15) and CCL4 were significantly associated with accelerated PAV, NCPV, and CPV progression. GDF-15 and CCL4 were identified as the most significantly associated proteins. GDF-15 showed a significant association with accelerated PAV and NCPV progression after correction for multiple testing, with coefficients of 0.076 (*P*_FDR_ = 0.007) and 0.114 (*P*_FDR_ = 0.007), respectively. CCL4 was significantly associated with increased PAV progression after multiple testing correction, evidenced by a coefficient of 0.084 (*P*_FDR_ = 0.013).

**Figure 3:**
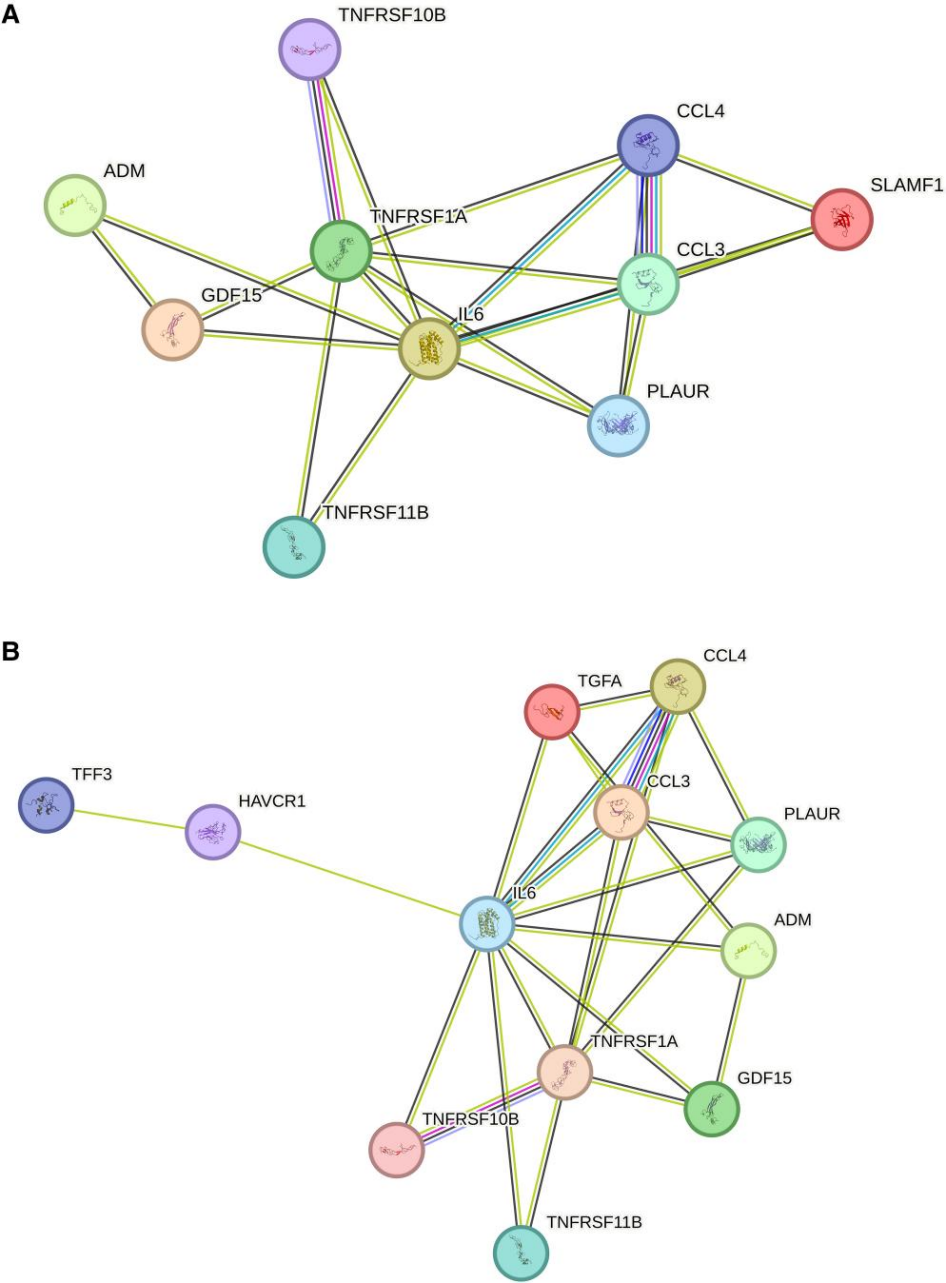
Protein network analysis. Protein network analyses of significant proteins related to (*A*) percent atheroma volume and (*B*) percent NCPV progression.

The PPI network for proteins involved in PAV progression identified 21 interactions with an enrichment *P* < 0.001 (*Figure 3A* and Supplementary data online, *Table S3*). The functional enrichment analysis substantiated the significance of these interactions, associating a total of 60 GO terms, detailed in Supplementary data online, *Table S4*. Similar patterns were observed in the NCPV progression analyses, with results detailed in *Figure 3B*; Supplementary data online, *Tables S3* and *S5*. The network and functional enrichment analyses identified key pathways across PAV and NCPV progression. These include an inflammatory pathway involving proteins CCL4, CCL3, TNF-R1, IL-6, GDF-15, and U-PAR; all important for mediating inflammatory responses (relevant GO terms: GO:0006954, GO:0050729, GO:0048585). Additionally, an apoptosis pathway was observed with proteins TRAIL-R2, TNF-R1, and GDF-15, which are linked to the regulation of programmed cell death in atherosclerosis (GO:0043065, GO:0042981, GO:0008625). Lastly, a chemotaxis and leukocyte activation pathway was observed, involving CCL4 and CCL3, which plays a significant role in recruiting immune cells to sites of vascular inflammation (GO:0002548, GO:0002690, GO:0030595).

## Discussion

This is the first study to demonstrate that integration of a targeted proteomic panel significantly improves prediction of long-term CCTA-defined plaque progression beyond traditional cardiovascular risk factors and baseline plaque volume (*Graphical Abstract*). The inclusion of a proteomic panel not only improved the prediction of long-term overall plaque progression, but also the prediction of the high-risk non-calcified plaque component as well as development of HRP. Collectively, these data underscore the promise of adding plasma biomarkers for identification of individuals with accelerated plaque progression and a HRP sub-type who have been shown to display a markedly higher risk for ASCVD events.

### Proteomic integration improves risk factor prediction for plaque progression

Plaque progression provides critical information for predicting future cardiovascular events. The PARADIGM study, which to the best of our knowledge is the largest study to date where serial CCTA imaging was performed, identified plaque progression, along with previous acute coronary syndrome (ACS), as the key variables independently associated with future cardiovascular events.^26^ In a consecutive study, the annualized increase in PAV was independently associated with major adverse cardiovascular events, with a 23% increased risk per SD PAV increase over 8 years of follow-up.^27^ The non-calcified plaque component, recognized as a more vulnerable plaque phenotype, was observed to be the plaque type predominantly responsible for the association with adverse clinical outcomes in this study.^27^ Although traditional risk factors such as diabetes and hypertension associate with plaque progression,^28,29^ considerable individual variability remains,^30–32^ showing that these markers fail to accurately identify patients with accelerated plaque progression. In support, it has been reported that 10–30% of ACS patients are hallmarked by the complete absence of traditional risk factors,^33–35^ emphasizing the need for improved prediction methods beyond traditional risk factors. The current study shows additional value of distinct plasma proteome panels in predicting plaque progression, surpassing traditional risk factors, and baseline plaque volume. We demonstrate that a distinct plasma proteomic panel can identify patients with accelerated PAV and NCPV progression, as well as HRP development during long-term follow-up. While the predictive accuracy of the clinical and clinical plus baseline plaque models were moderate with AUCs ranging from 0.7 to 0.8, integrating a plasma proteomic panel with the former parameters significantly enhanced this accuracy, achieving AUCs of ∼0.8 to 0.9. The approach where proteomics is added in the prediction model could enable the identification of high-risk patients without the need for repeated CCTAs. For patients with coronary plaques, this ‘liquid health check’ could serve as a non-invasive alternative for monitoring plaque progression.^36^ Tentatively, this approach would allow for more frequent and accessible assessments, reducing the costs, burden, and potential risks associated with repeated imaging procedures.

### Proteins associated with accelerated plaque progression

The proteins associating with accelerated plaque progression merit further consideration aiming to unravel underlying pathophysiological processes. The most important individual proteins in our study, significant after multiple testing correction, were GDF-15 and CCL4. GDF-15, a stress-responsive member of the transforming growth factor-β (TGF-β) cytokine superfamily, has been shown to be a strong and independent predictor of cardiovascular events.^37,38^ Although the exact biological functions of GDF-15 are still not completely understood, it has been shown to regulate inflammatory, angiogenesis and apoptotic pathways.^39^ CCL4, also known as macrophage inflammatory protein-1β, is a member of the CC chemokine family and has a complex mediating role in immune responses.^40^ Inhibition of CCL4 by antibody treatment in a mice model was shown to decrease both vascular inflammation, and plaque area, in conjunction with a beneficial effect on plaque stability.^41^

A number of proteins were found to be associated with adverse plaque phenotypes in previous cross-sectional studies, and it is noteworthy that their findings do not consistently align with those of the current study. In our previous study in the same cohort, a total of 35 proteins were shown to be associated with HRP features by means of a machine learning model.^12^ Matrix metalloproteinase 12 (MMP-12) was identified among the significant proteins associated with HRP development in our study. Another study correlated HRP presence with plasma proteomics utilizing four Olink panels; three of which were also analysed in our study.^42^ In this study, 10 proteins were significantly associated with HRP presence in univariable analyses after multiple-testing correction, but none overlapped with those identified in our study. Another study examined the relationship between 5000 proteins, measured with an aptamer-based assay, and CCTA-derived plaque presence.^43^ The proteins GDF-15, TRAIL-R2, and TNF-R2, identified in this study, were consistent with those found in our research. The discrepancies across studies may be attributed to differences in patient characteristics, the CCTA scanners used, and the proteins and platforms selected.

### Inflammatory pathways associated with plaque progression

Pathway analyses showed key pathways related to inflammation, apoptosis, and chemotaxis, along with leukocyte activation. Endothelial activation initiates the recruitment and accumulation of lipid and immune cells within the vasculature.^44^ During these initial stages of atherogenesis, both chemotaxis of immune cells and production of pro-inflammatory cytokines are critical.^45^ CCL3 and CCL4 have been observed to be secreted by activated macrophages, facilitating the chemotaxis of immune cells.^40,41^ As the disease progresses, monocytes and smooth muscle cells may not only internalize lipid particles but also amplify pro-inflammatory signalling, thereby accelerating plaque development. A central pro-inflammatory role has been identified for IL-6, a cytokine within the NLRP3 inflammasome pathway, which increases vascular permeability, stimulates endothelial cells and recruits immune cells.^46^ Furthermore, pro-apoptotic proteins such as TRAIL-R2 and its receptor OPG, TNF-R1, and GDF-15 have been observed to contribute to plaque instability and rupture.^39,47–49^

The progression related pathways of inflammation, chemotaxis, and apoptosis are integral throughout all stages of plaque development and progression. Despite the higher vulnerability of the non-calcified plaque type, we found no substantial differences in the proteins related to overall plaque progression. This suggests that therapeutic strategies aimed at targeting plaque progression could focus on these common proteins and pathways, potentially offering a unified approach that does not require differentiation between these plaque types.

### Clinical implications

The use of CCTA has proven effective in diagnosing coronary heart disease and has led to changes in management strategies,^50^ resulting in reduced cardiovascular events primarily due to increased preventive treatment prescriptions.^51^ Beyond evaluating a single CCTA scan, identifying accelerated plaque progression offers the potential to better ascertain patients at the highest risk. These patients predominantly benefit from aggressive treatment, which has consistently been shown to halt and even reverse plaque progression.^52–54^ Considering the high costs of novel medications^55^ and the persistent problem of low adherence to clinical guidelines,^56,57^ it is crucial to identify high(est)-risk individuals who would benefit most from personalized and intensified therapeutic regimens. This study demonstrates that plasma proteomics can aid in identifying these high(est)-risk individuals, potentially precluding the need for repeated CCTA’s. Further research is needed to fully evaluate the impact of proteomics in identifying plaque progression and its potential to improve patient outcomes.

### Strength and limitations

The study was limited by a small sample size, which reduced the statistical power of the models and precluded defining accelerated plaque progression beyond using the median for PAV, NCPV, and CPV. The median PAV progression of 1.17% over an 8-year follow-up period was relatively low, which may affect the generalizability of our findings to patients with more unstable disease. While the proteomic analyses were conducted using a validated targeted panel of proteins related to cardiovascular disease, contemporary panels that estimate a broader spectrum of circulating proteins could potentially strengthen and validate the observations more effectively. While the absence of a validation cohort is an important limitation, the corrected AUCs demonstrated low levels of optimism, which supports the robustness of the observations in our study. A key strength of this study is the long-term follow-up period between the CCTA derived measures, which significantly enhances the robustness of our findings.

## Conclusions

This study demonstrates that integrating a targeted proteomic panel significantly enhances the prediction of long-term CCTA-defined plaque progression beyond traditional cardiovascular risk factors and baseline plaque volume. The inclusion of proteomics not only improves overall plaque progression prediction but also identifies high-risk non-calcified plaques and HRP development. These findings highlight the potential of plasma biomarkers in identifying individuals at increased risk for ASCVD events, which could enable more precise and personalized treatment strategies while reducing the need for repeated CCTAs.

## Supplementary Material

jeae313_Supplementary_Data

## Data Availability

All data are available from the corresponding author upon reasonable request.
