# Supplementary material for: Plasma proteomics improves prediction of coronary plaque progression
Source: Eur Heart J Cardiovasc Imaging. 2024 Dec 10;26(3):489–99. doi: 10.1093/ehjci/jeae313 (PMC11879226; doi:10.1093/ehjci/jeae313)
Supplement: jeae313_Supplementary_Data [file jeae313_supplementary_data.docx]

**Table S1. Baseline and follow-up CCTA plaque measures**

|  | Baseline | Follow-up | Difference |
| --- | --- | --- | --- |
| Percent atheroma volume (%) | 2.94 [0.74, 8.16] | 6.52 [1.43, 12.40] | 1.17 [0.29, 4.08] |
| Percent noncalcified plaque volume (%) | 2.10 [0.59, 4.95] | 3.20 [0.96, 6.98] | 0.45 [-0.07, 2.27] |
| Percent calcified plaques (%) | 0.85 [0.00, 2.28] | 1.81 [0.09, 5.13] | 0.58 [0.21, 2.05] |
| High-risk plaque (n) | 43 (44.3%) | 47 (48.5%) | - |

Median [IQR] levels of baseline, follow-up and difference for percent atheroma plaque volume, percent noncalcified plaque volume, percent calcified plaque volume and high-risk plaque development.

**Table S2. Clinical and imaging-based predictors of PAV progression**

| **Characteristics** | **Univariable model** Beta coefficient | **p-value** | **Age- and sex-adjusted model** Beta coefficient | **p-value** |
| --- | --- | --- | --- | --- |
| Age (per 5 years) | 0.270 | 0.216 |  |  |
| Male sex | 0.119 | 0.861 |  |  |
| Current smoker (yes/no) | -0.948 | 0.234 | -0.866 | 0.290 |
| Diabetes mellitus (yes/no) | 1.936 | **0.028** | 1.914 | **0.034** |
| Hypertension (yes/no) | 0.821 | 0.206 | 0.775 | 0.255 |
| Hypercholesterolemia (yes/no) | 0.004 | 0.815 | 0.002 | 0.879 |
| Family history of CAD (yes/no) | -0.456 | 0.481 | -0.347 | 0.608 |
| BMI (kg/m^2^) | 0.089 | 0.293 | 0.095 | 0.265 |
| Statin intensity | 0.367 | 0.245 | 0.339 | 0.286 |
| Beta blockers use (yes/no) | -0.299 | 0.651 | -0.383 | 0.566 |
| Aspirin use (yes/no) | 0.399 | 0.664 | 0.231 | 0.804 |
| Calcium blockers use (yes/no) | 0.970 | 0.970 | 0.829 | 0.264 |
| Total cholesterol (per 1 mmol/L) | -0.139 | 0.658 | -0.025 | 0.939 |
| LDL cholesterol (per 1mmol/L) | -0.216 | 0.518 | -0.096 | 0.786 |
| HDL cholesterol (per 1 mmol/L) | 0.267 | 0.663 | 0.317 | 0.614 |
| Triglycerides (per 1 mmol/L) | 0.454 | 0.279 | 0.028 | 0.260 |
| Lipoprotein(a) (per 25 nmol/L) | 0.120 | 0.170 | 0.122 | 0.164 |
| eGFR (per 10 mL/min/1.73m2) | 0.063 | 0.683 | 0.146 | 0.439 |
| PAV at baseline (per 1%) | 0.119 | **0.014** | 0.112 | **0.023** |
| NCPV at baseline (per 1%) | 0.003 | 0.306 | 0.003 | 0.389 |
| CPV at baseline (per 1%) | 0.006 | 0.081 | 0.006 | 0.126 |
| HRP at baseline | 1.476 | **0.022** | 1.692 | **0.016** |

Association of clinical and imaging parameters with PAV progression over the study period. Linear regression models were applied in univariable analyses (left) and in age- and sex-adjusted models (right). BMI: Body Mass Index, LDL: Low-density lipoprotein, HDL: high-density lipoprotein, eGFR: estimated Glomerular Filtration Rate, PAV: percent atheroma volume. NCPV: percent noncalcified percent volume, CPV: percent calcified volume, HRP: high-risk plaque.

|  | Number of edges | Average node degree | Local clustering coefficient | PPI enrichment p-value |
| --- | --- | --- | --- | --- |
| Percent atheroma volume | 21 | 4.2 | 0.803 | <0.001 |
| Percent noncalcified plaque volume | 20 | 3.33 | 0.698 | <0.001 |

**Table S3. Network analysis significant proteins**

Network analysis using the STRING database for significant proteins for percent atheroma value and percent noncalcified plaque volume. Shown are the statistics for number of edges, representing the total connections between proteins, average node degree, indicating the average number of connections per protein, local clustering coefficient, measuring the degree to which proteins tend to cluster together, and the PPI enrichment p-value, assessing the statistical significance of the protein interactions being more connected than expected by chance.

**Table S4. Biological Process PAV progression**

| **#term ID** | **term description** | **observed gene count** | **background gene count** | **strength** | **false discovery rate** | **matching proteins in your network (IDs)** | **matching proteins in your network (labels)** |
| --- | --- | --- | --- | --- | --- | --- | --- |
| GO:0009605 | Response to external stimulus | 10 | 2355 | 0.92 | 9.55e-06 | 9606.ENSP00000162749,9606.ENSP00000252809,9606.ENSP00000276431,9606.ENSP00000297350,9606.ENSP00000339328,9606.ENSP00000385675,9606.ENSP00000436607,9606.ENSP00000438406,9606.ENSP00000477908,9606.ENSP00000482259 | TNFRSF1A,GDF15,TNFRSF10B,TNFRSF11B,PLAUR,IL6,ADM,SLAMF1,CCL3,CCL4 |
| GO:0009967 | Positive regulation of signal transduction | 8 | 1525 | 1.01 | 0.00040 | 9606.ENSP00000162749,9606.ENSP00000252809,9606.ENSP00000276431,9606.ENSP00000339328,9606.ENSP00000385675,9606.ENSP00000438406,9606.ENSP00000477908,9606.ENSP00000482259 | TNFRSF1A,GDF15,TNFRSF10B,PLAUR,IL6,SLAMF1,CCL3,CCL4 |
| GO:0002690 | Positive regulation of leukocyte chemotaxis | 4 | 99 | 1.9 | 0.00043 | 9606.ENSP00000385675,9606.ENSP00000438406,9606.ENSP00000477908,9606.ENSP00000482259 | IL6,SLAMF1,CCL3,CCL4 |
| GO:0006954 | Inflammatory response | 6 | 538 | 1.34 | 0.00043 | 9606.ENSP00000162749,9606.ENSP00000385675,9606.ENSP00000436607,9606.ENSP00000438406,9606.ENSP00000477908,9606.ENSP00000482259 | TNFRSF1A,IL6,ADM,SLAMF1,CCL3,CCL4 |
| GO:0051241 | Negative regulation of multicellular organismal process | 7 | 1035 | 1.12 | 0.00043 | 9606.ENSP00000162749,9606.ENSP00000252809,9606.ENSP00000297350,9606.ENSP00000385675,9606.ENSP00000436607,9606.ENSP00000438406,9606.ENSP00000477908 | TNFRSF1A,GDF15,TNFRSF11B,IL6,ADM,SLAMF1,CCL3 |
| GO:1902531 | Regulation of intracellular signal transduction | 8 | 1726 | 0.96 | 0.00043 | 9606.ENSP00000162749,9606.ENSP00000252809,9606.ENSP00000276431,9606.ENSP00000339328,9606.ENSP00000385675,9606.ENSP00000438406,9606.ENSP00000477908,9606.ENSP00000482259 | TNFRSF1A,GDF15,TNFRSF10B,PLAUR,IL6,SLAMF1,CCL3,CCL4 |
| GO:1902533 | Positive regulation of intracellular signal transduction | 7 | 997 | 1.14 | 0.00043 | 9606.ENSP00000162749,9606.ENSP00000252809,9606.ENSP00000276431,9606.ENSP00000385675,9606.ENSP00000438406,9606.ENSP00000477908,9606.ENSP00000482259 | TNFRSF1A,GDF15,TNFRSF10B,IL6,SLAMF1,CCL3,CCL4 |
| GO:0009966 | Regulation of signal transduction | 9 | 2978 | 0.77 | 0.00057 | 9606.ENSP00000162749,9606.ENSP00000252809,9606.ENSP00000276431,9606.ENSP00000339328,9606.ENSP00000385675,9606.ENSP00000436607,9606.ENSP00000438406,9606.ENSP00000477908,9606.ENSP00000482259 | TNFRSF1A,GDF15,TNFRSF10B,PLAUR,IL6,ADM,SLAMF1,CCL3,CCL4 |
| GO:0006952 | Defense response | 7 | 1394 | 1.0 | 0.00088 | 9606.ENSP00000162749,9606.ENSP00000276431,9606.ENSP00000385675,9606.ENSP00000436607,9606.ENSP00000438406,9606.ENSP00000477908,9606.ENSP00000482259 | TNFRSF1A,TNFRSF10B,IL6,ADM,SLAMF1,CCL3,CCL4 |
| GO:0009607 | Response to biotic stimulus | 7 | 1375 | 1.0 | 0.00088 | 9606.ENSP00000162749,9606.ENSP00000276431,9606.ENSP00000385675,9606.ENSP00000436607,9606.ENSP00000438406,9606.ENSP00000477908,9606.ENSP00000482259 | TNFRSF1A,TNFRSF10B,IL6,ADM,SLAMF1,CCL3,CCL4 |
| GO:0030595 | Leukocyte chemotaxis | 4 | 149 | 1.72 | 0.00088 | 9606.ENSP00000385675,9606.ENSP00000438406,9606.ENSP00000477908,9606.ENSP00000482259 | IL6,SLAMF1,CCL3,CCL4 |
| GO:0002548 | Monocyte chemotaxis | 3 | 43 | 2.14 | 0.0011 | 9606.ENSP00000385675,9606.ENSP00000477908,9606.ENSP00000482259 | IL6,CCL3,CCL4 |
| GO:0032103 | Positive regulation of response to external stimulus | 5 | 453 | 1.34 | 0.0011 | 9606.ENSP00000162749,9606.ENSP00000385675,9606.ENSP00000438406,9606.ENSP00000477908,9606.ENSP00000482259 | TNFRSF1A,IL6,SLAMF1,CCL3,CCL4 |
| GO:0043410 | Positive regulation of MAPK cascade | 5 | 481 | 1.31 | 0.0014 | 9606.ENSP00000252809,9606.ENSP00000385675,9606.ENSP00000438406,9606.ENSP00000477908,9606.ENSP00000482259 | GDF15,IL6,SLAMF1,CCL3,CCL4 |
| GO:0006935 | Chemotaxis | 5 | 516 | 1.28 | 0.0017 | 9606.ENSP00000339328,9606.ENSP00000385675,9606.ENSP00000438406,9606.ENSP00000477908,9606.ENSP00000482259 | PLAUR,IL6,SLAMF1,CCL3,CCL4 |
| GO:0032101 | Regulation of response to external stimulus | 6 | 964 | 1.09 | 0.0017 | 9606.ENSP00000162749,9606.ENSP00000339328,9606.ENSP00000385675,9606.ENSP00000438406,9606.ENSP00000477908,9606.ENSP00000482259 | TNFRSF1A,PLAUR,IL6,SLAMF1,CCL3,CCL4 |
| GO:0043065 | Positive regulation of apoptotic process | 5 | 507 | 1.29 | 0.0017 | 9606.ENSP00000162749,9606.ENSP00000276431,9606.ENSP00000385675,9606.ENSP00000436607,9606.ENSP00000477908 | TNFRSF1A,TNFRSF10B,IL6,ADM,CCL3 |
| GO:0098542 | Defense response to other organism | 6 | 989 | 1.08 | 0.0017 | 9606.ENSP00000162749,9606.ENSP00000385675,9606.ENSP00000436607,9606.ENSP00000438406,9606.ENSP00000477908,9606.ENSP00000482259 | TNFRSF1A,IL6,ADM,SLAMF1,CCL3,CCL4 |
| GO:0042221 | Response to chemical | 9 | 4010 | 0.65 | 0.0023 | 9606.ENSP00000162749,9606.ENSP00000252809,9606.ENSP00000297350,9606.ENSP00000339328,9606.ENSP00000385675,9606.ENSP00000436607,9606.ENSP00000438406,9606.ENSP00000477908,9606.ENSP00000482259 | TNFRSF1A,GDF15,TNFRSF11B,PLAUR,IL6,ADM,SLAMF1,CCL3,CCL4 |
| GO:0051239 | Regulation of multicellular organismal process | 8 | 2749 | 0.76 | 0.0023 | 9606.ENSP00000162749,9606.ENSP00000252809,9606.ENSP00000297350,9606.ENSP00000339328,9606.ENSP00000385675,9606.ENSP00000436607,9606.ENSP00000438406,9606.ENSP00000477908 | TNFRSF1A,GDF15,TNFRSF11B,PLAUR,IL6,ADM,SLAMF1,CCL3 |
| GO:2000503 | Positive regulation of natural killer cell chemotaxis | 2 | 6 | 2.82 | 0.0027 | 9606.ENSP00000477908,9606.ENSP00000482259 | CCL3,CCL4 |
| GO:0071677 | Positive regulation of mononuclear cell migration | 3 | 75 | 1.9 | 0.0028 | 9606.ENSP00000438406,9606.ENSP00000477908,9606.ENSP00000482259 | SLAMF1,CCL3,CCL4 |
| GO:0007166 | Cell surface receptor signaling pathway | 7 | 2040 | 0.83 | 0.0044 | 9606.ENSP00000162749,9606.ENSP00000252809,9606.ENSP00000276431,9606.ENSP00000339328,9606.ENSP00000385675,9606.ENSP00000477908,9606.ENSP00000482259 | TNFRSF1A,GDF15,TNFRSF10B,PLAUR,IL6,CCL3,CCL4 |
| GO:0051716 | Cellular response to stimulus | 10 | 6357 | 0.49 | 0.0045 | 9606.ENSP00000162749,9606.ENSP00000252809,9606.ENSP00000276431,9606.ENSP00000297350,9606.ENSP00000339328,9606.ENSP00000385675,9606.ENSP00000436607,9606.ENSP00000438406,9606.ENSP00000477908,9606.ENSP00000482259 | TNFRSF1A,GDF15,TNFRSF10B,TNFRSF11B,PLAUR,IL6,ADM,SLAMF1,CCL3,CCL4 |
| GO:0001934 | Positive regulation of protein phosphorylation | 5 | 747 | 1.12 | 0.0058 | 9606.ENSP00000162749,9606.ENSP00000252809,9606.ENSP00000276431,9606.ENSP00000339328,9606.ENSP00000385675 | TNFRSF1A,GDF15,TNFRSF10B,PLAUR,IL6 |
| GO:0007165 | Signal transduction | 9 | 4714 | 0.58 | 0.0068 | 9606.ENSP00000162749,9606.ENSP00000252809,9606.ENSP00000276431,9606.ENSP00000297350,9606.ENSP00000339328,9606.ENSP00000385675,9606.ENSP00000436607,9606.ENSP00000477908,9606.ENSP00000482259 | TNFRSF1A,GDF15,TNFRSF10B,TNFRSF11B,PLAUR,IL6,ADM,CCL3,CCL4 |
| GO:0006950 | Response to stress | 8 | 3358 | 0.67 | 0.0076 | 9606.ENSP00000162749,9606.ENSP00000276431,9606.ENSP00000339328,9606.ENSP00000385675,9606.ENSP00000436607,9606.ENSP00000438406,9606.ENSP00000477908,9606.ENSP00000482259 | TNFRSF1A,TNFRSF10B,PLAUR,IL6,ADM,SLAMF1,CCL3,CCL4 |
| GO:0019221 | Cytokine-mediated signaling pathway | 4 | 369 | 1.33 | 0.0078 | 9606.ENSP00000162749,9606.ENSP00000385675,9606.ENSP00000477908,9606.ENSP00000482259 | TNFRSF1A,IL6,CCL3,CCL4 |
| GO:0042981 | Regulation of apoptotic process | 6 | 1462 | 0.91 | 0.0084 | 9606.ENSP00000162749,9606.ENSP00000276431,9606.ENSP00000339328,9606.ENSP00000385675,9606.ENSP00000436607,9606.ENSP00000477908 | TNFRSF1A,TNFRSF10B,PLAUR,IL6,ADM,CCL3 |
| GO:0043922 | Negative regulation by host of viral transcription | 2 | 15 | 2.42 | 0.0090 | 9606.ENSP00000477908,9606.ENSP00000482259 | CCL3,CCL4 |
| GO:0150078 | Positive regulation of neuroinflammatory response | 2 | 15 | 2.42 | 0.0090 | 9606.ENSP00000385675,9606.ENSP00000477908 | IL6,CCL3 |
| GO:0034112 | Positive regulation of homotypic cell-cell adhesion | 2 | 17 | 2.37 | 0.0105 | 9606.ENSP00000339328,9606.ENSP00000385675 | PLAUR,IL6 |
| GO:0045779 | Negative regulation of bone resorption | 2 | 17 | 2.37 | 0.0105 | 9606.ENSP00000297350,9606.ENSP00000385675 | TNFRSF11B,IL6 |
| GO:0048245 | Eosinophil chemotaxis | 2 | 17 | 2.37 | 0.0105 | 9606.ENSP00000477908,9606.ENSP00000482259 | CCL3,CCL4 |
| GO:1903978 | Regulation of microglial cell activation | 2 | 18 | 2.34 | 0.0109 | 9606.ENSP00000385675,9606.ENSP00000477908 | IL6,CCL3 |
| GO:0048585 | Negative regulation of response to stimulus | 6 | 1612 | 0.87 | 0.0110 | 9606.ENSP00000162749,9606.ENSP00000252809,9606.ENSP00000339328,9606.ENSP00000385675,9606.ENSP00000436607,9606.ENSP00000438406 | TNFRSF1A,GDF15,PLAUR,IL6,ADM,SLAMF1 |
| GO:0050729 | Positive regulation of inflammatory response | 3 | 145 | 1.61 | 0.0110 | 9606.ENSP00000162749,9606.ENSP00000385675,9606.ENSP00000477908 | TNFRSF1A,IL6,CCL3 |
| GO:0009893 | Positive regulation of metabolic process | 8 | 3847 | 0.61 | 0.0141 | 9606.ENSP00000162749,9606.ENSP00000252809,9606.ENSP00000276431,9606.ENSP00000339328,9606.ENSP00000385675,9606.ENSP00000436607,9606.ENSP00000438406,9606.ENSP00000477908 | TNFRSF1A,GDF15,TNFRSF10B,PLAUR,IL6,ADM,SLAMF1,CCL3 |
| GO:0032680 | Regulation of tumor necrosis factor production | 3 | 164 | 1.56 | 0.0145 | 9606.ENSP00000385675,9606.ENSP00000438406,9606.ENSP00000477908 | IL6,SLAMF1,CCL3 |
| GO:0010033 | Response to organic substance | 7 | 2692 | 0.71 | 0.0154 | 9606.ENSP00000162749,9606.ENSP00000252809,9606.ENSP00000297350,9606.ENSP00000385675,9606.ENSP00000436607,9606.ENSP00000477908,9606.ENSP00000482259 | TNFRSF1A,GDF15,TNFRSF11B,IL6,ADM,CCL3,CCL4 |
| GO:0071356 | Cellular response to tumor necrosis factor | 3 | 175 | 1.53 | 0.0165 | 9606.ENSP00000162749,9606.ENSP00000477908,9606.ENSP00000482259 | TNFRSF1A,CCL3,CCL4 |
| GO:0002726 | Positive regulation of T cell cytokine production | 2 | 26 | 2.18 | 0.0170 | 9606.ENSP00000385675,9606.ENSP00000438406 | IL6,SLAMF1 |
| GO:0048522 | Positive regulation of cellular process | 9 | 5584 | 0.5 | 0.0171 | 9606.ENSP00000162749,9606.ENSP00000252809,9606.ENSP00000276431,9606.ENSP00000339328,9606.ENSP00000385675,9606.ENSP00000436607,9606.ENSP00000438406,9606.ENSP00000477908,9606.ENSP00000482259 | TNFRSF1A,GDF15,TNFRSF10B,PLAUR,IL6,ADM,SLAMF1,CCL3,CCL4 |
| GO:1905523 | Positive regulation of macrophage migration | 2 | 27 | 2.16 | 0.0179 | 9606.ENSP00000438406,9606.ENSP00000477908 | SLAMF1,CCL3 |
| GO:0007267 | Cell-cell signaling | 5 | 1079 | 0.96 | 0.0184 | 9606.ENSP00000252809,9606.ENSP00000385675,9606.ENSP00000436607,9606.ENSP00000477908,9606.ENSP00000482259 | GDF15,IL6,ADM,CCL3,CCL4 |
| GO:0002366 | Leukocyte activation involved in immune response | 3 | 190 | 1.49 | 0.0190 | 9606.ENSP00000385675,9606.ENSP00000438406,9606.ENSP00000477908 | IL6,SLAMF1,CCL3 |
| GO:0045671 | Negative regulation of osteoclast differentiation | 2 | 33 | 2.08 | 0.0230 | 9606.ENSP00000297350,9606.ENSP00000477908 | TNFRSF11B,CCL3 |
| GO:0070374 | Positive regulation of ERK1 and ERK2 cascade | 3 | 213 | 1.44 | 0.0243 | 9606.ENSP00000438406,9606.ENSP00000477908,9606.ENSP00000482259 | SLAMF1,CCL3,CCL4 |
| GO:0009968 | Negative regulation of signal transduction | 5 | 1252 | 0.9 | 0.0321 | 9606.ENSP00000252809,9606.ENSP00000339328,9606.ENSP00000385675,9606.ENSP00000436607,9606.ENSP00000438406 | GDF15,PLAUR,IL6,ADM,SLAMF1 |
| GO:0050850 | Positive regulation of calcium-mediated signaling | 2 | 42 | 1.97 | 0.0340 | 9606.ENSP00000477908,9606.ENSP00000482259 | CCL3,CCL4 |
| GO:0008625 | Extrinsic apoptotic signaling pathway via death domain receptors | 2 | 43 | 1.96 | 0.0349 | 9606.ENSP00000162749,9606.ENSP00000276431 | TNFRSF1A,TNFRSF10B |
| GO:0009617 | Response to bacterium | 4 | 663 | 1.07 | 0.0349 | 9606.ENSP00000162749,9606.ENSP00000385675,9606.ENSP00000436607,9606.ENSP00000477908 | TNFRSF1A,IL6,ADM,CCL3 |
| GO:0044403 | Biological process involved in symbiotic interaction | 3 | 257 | 1.36 | 0.0373 | 9606.ENSP00000438406,9606.ENSP00000477908,9606.ENSP00000482259 | SLAMF1,CCL3,CCL4 |
| GO:0006955 | Immune response | 5 | 1321 | 0.87 | 0.0376 | 9606.ENSP00000385675,9606.ENSP00000436607,9606.ENSP00000438406,9606.ENSP00000477908,9606.ENSP00000482259 | IL6,ADM,SLAMF1,CCL3,CCL4 |
| GO:0048247 | Lymphocyte chemotaxis | 2 | 51 | 1.89 | 0.0431 | 9606.ENSP00000477908,9606.ENSP00000482259 | CCL3,CCL4 |
| GO:0048523 | Negative regulation of cellular process | 8 | 4736 | 0.52 | 0.0431 | 9606.ENSP00000162749,9606.ENSP00000252809,9606.ENSP00000297350,9606.ENSP00000339328,9606.ENSP00000385675,9606.ENSP00000436607,9606.ENSP00000438406,9606.ENSP00000477908 | TNFRSF1A,GDF15,TNFRSF11B,PLAUR,IL6,ADM,SLAMF1,CCL3 |
| GO:0080134 | Regulation of response to stress | 5 | 1373 | 0.86 | 0.0431 | 9606.ENSP00000162749,9606.ENSP00000339328,9606.ENSP00000385675,9606.ENSP00000438406,9606.ENSP00000477908 | TNFRSF1A,PLAUR,IL6,SLAMF1,CCL3 |
| GO:2000026 | Regulation of multicellular organismal development | 5 | 1389 | 0.85 | 0.0443 | 9606.ENSP00000162749,9606.ENSP00000297350,9606.ENSP00000385675,9606.ENSP00000436607,9606.ENSP00000477908 | TNFRSF1A,TNFRSF11B,IL6,ADM,CCL3 |
| GO:0002682 | Regulation of immune system process | 5 | 1438 | 0.84 | 0.0493 | 9606.ENSP00000297350,9606.ENSP00000385675,9606.ENSP00000438406,9606.ENSP00000477908,9606.ENSP00000482259 | TNFRSF11B,IL6,SLAMF1,CCL3,CCL4 |
| GO:1903053 | Regulation of extracellular matrix organization | 2 | 56 | 1.85 | 0.0493 | 9606.ENSP00000162749,9606.ENSP00000385675 | TNFRSF1A,IL6 |

Significantly associated biological processes (Gene Ontology) with accelerated PAV progression (n=60). PAV: percent atheroma volume.

**Table S5. Biological Process NCPV progression**

| **#term ID** | **term description** | **observed gene count** | **background gene count** | **strength** | **false discovery rate** | **matching proteins in your network (IDs)** | **matching proteins in your network (labels)** |
| --- | --- | --- | --- | --- | --- | --- | --- |
| GO:0009605 | Response to external stimulus | 9 | 2355 | 0.8 | 0.0111 | 9606.ENSP00000162749,9606.ENSP00000252809,9606.ENSP00000276431,9606.ENSP00000297350,9606.ENSP00000339328,9606.ENSP00000385675,9606.ENSP00000436607,9606.ENSP00000477908,9606.ENSP00000482259 | TNFRSF1A,GDF15,TNFRSF10B,TNFRSF11B,PLAUR,IL6,ADM,CCL3,CCL4 |
| GO:0051241 | Negative regulation of multicellular organismal process | 7 | 1035 | 1.05 | 0.0111 | 9606.ENSP00000162749,9606.ENSP00000252809,9606.ENSP00000297350,9606.ENSP00000384126,9606.ENSP00000385675,9606.ENSP00000436607,9606.ENSP00000477908 | TNFRSF1A,GDF15,TNFRSF11B,LRRC32,IL6,ADM,CCL3 |
| GO:0002548 | Monocyte chemotaxis | 3 | 43 | 2.06 | 0.0135 | 9606.ENSP00000385675,9606.ENSP00000477908,9606.ENSP00000482259 | IL6,CCL3,CCL4 |
| GO:0007166 | Cell surface receptor signaling pathway | 8 | 2040 | 0.81 | 0.0177 | 9606.ENSP00000162749,9606.ENSP00000252809,9606.ENSP00000276431,9606.ENSP00000339328,9606.ENSP00000384126,9606.ENSP00000385675,9606.ENSP00000477908,9606.ENSP00000482259 | TNFRSF1A,GDF15,TNFRSF10B,PLAUR,LRRC32,IL6,CCL3,CCL4 |
| GO:0002690 | Positive regulation of leukocyte chemotaxis | 3 | 99 | 1.7 | 0.0248 | 9606.ENSP00000385675,9606.ENSP00000477908,9606.ENSP00000482259 | IL6,CCL3,CCL4 |
| GO:0006954 | Inflammatory response | 5 | 538 | 1.18 | 0.0248 | 9606.ENSP00000162749,9606.ENSP00000385675,9606.ENSP00000436607,9606.ENSP00000477908,9606.ENSP00000482259 | TNFRSF1A,IL6,ADM,CCL3,CCL4 |
| GO:0007165 | Signal transduction | 10 | 4714 | 0.54 | 0.0248 | 9606.ENSP00000162749,9606.ENSP00000252809,9606.ENSP00000276431,9606.ENSP00000297350,9606.ENSP00000339328,9606.ENSP00000384126,9606.ENSP00000385675,9606.ENSP00000436607,9606.ENSP00000477908,9606.ENSP00000482259 | TNFRSF1A,GDF15,TNFRSF10B,TNFRSF11B,PLAUR,LRRC32,IL6,ADM,CCL3,CCL4 |
| GO:0009967 | Positive regulation of signal transduction | 7 | 1525 | 0.88 | 0.0248 | 9606.ENSP00000162749,9606.ENSP00000252809,9606.ENSP00000276431,9606.ENSP00000339328,9606.ENSP00000385675,9606.ENSP00000477908,9606.ENSP00000482259 | TNFRSF1A,GDF15,TNFRSF10B,PLAUR,IL6,CCL3,CCL4 |
| GO:0010646 | Regulation of cell communication | 9 | 3355 | 0.64 | 0.0248 | 9606.ENSP00000162749,9606.ENSP00000252809,9606.ENSP00000276431,9606.ENSP00000339328,9606.ENSP00000384126,9606.ENSP00000385675,9606.ENSP00000436607,9606.ENSP00000477908,9606.ENSP00000482259 | TNFRSF1A,GDF15,TNFRSF10B,PLAUR,LRRC32,IL6,ADM,CCL3,CCL4 |
| GO:0023051 | Regulation of signaling | 9 | 3367 | 0.64 | 0.0248 | 9606.ENSP00000162749,9606.ENSP00000252809,9606.ENSP00000276431,9606.ENSP00000339328,9606.ENSP00000384126,9606.ENSP00000385675,9606.ENSP00000436607,9606.ENSP00000477908,9606.ENSP00000482259 | TNFRSF1A,GDF15,TNFRSF10B,PLAUR,LRRC32,IL6,ADM,CCL3,CCL4 |
| GO:0043065 | Positive regulation of apoptotic process | 5 | 507 | 1.21 | 0.0248 | 9606.ENSP00000162749,9606.ENSP00000276431,9606.ENSP00000385675,9606.ENSP00000436607,9606.ENSP00000477908 | TNFRSF1A,TNFRSF10B,IL6,ADM,CCL3 |
| GO:1902531 | Regulation of intracellular signal transduction | 7 | 1726 | 0.82 | 0.0248 | 9606.ENSP00000162749,9606.ENSP00000252809,9606.ENSP00000276431,9606.ENSP00000339328,9606.ENSP00000385675,9606.ENSP00000477908,9606.ENSP00000482259 | TNFRSF1A,GDF15,TNFRSF10B,PLAUR,IL6,CCL3,CCL4 |
| GO:1902533 | Positive regulation of intracellular signal transduction | 6 | 997 | 0.99 | 0.0248 | 9606.ENSP00000162749,9606.ENSP00000252809,9606.ENSP00000276431,9606.ENSP00000385675,9606.ENSP00000477908,9606.ENSP00000482259 | TNFRSF1A,GDF15,TNFRSF10B,IL6,CCL3,CCL4 |
| GO:2000503 | Positive regulation of natural killer cell chemotaxis | 2 | 6 | 2.74 | 0.0248 | 9606.ENSP00000477908,9606.ENSP00000482259 | CCL3,CCL4 |
| GO:0010033 | Response to organic substance | 8 | 2692 | 0.69 | 0.0284 | 9606.ENSP00000162749,9606.ENSP00000252809,9606.ENSP00000297350,9606.ENSP00000384126,9606.ENSP00000385675,9606.ENSP00000436607,9606.ENSP00000477908,9606.ENSP00000482259 | TNFRSF1A,GDF15,TNFRSF11B,LRRC32,IL6,ADM,CCL3,CCL4 |
| GO:0051239 | Regulation of multicellular organismal process | 8 | 2749 | 0.68 | 0.0317 | 9606.ENSP00000162749,9606.ENSP00000252809,9606.ENSP00000297350,9606.ENSP00000339328,9606.ENSP00000384126,9606.ENSP00000385675,9606.ENSP00000436607,9606.ENSP00000477908 | TNFRSF1A,GDF15,TNFRSF11B,PLAUR,LRRC32,IL6,ADM,CCL3 |
| GO:0001934 | Positive regulation of protein phosphorylation | 5 | 747 | 1.04 | 0.0323 | 9606.ENSP00000162749,9606.ENSP00000252809,9606.ENSP00000276431,9606.ENSP00000339328,9606.ENSP00000385675 | TNFRSF1A,GDF15,TNFRSF10B,PLAUR,IL6 |
| GO:0019221 | Cytokine-mediated signaling pathway | 4 | 369 | 1.25 | 0.0323 | 9606.ENSP00000162749,9606.ENSP00000385675,9606.ENSP00000477908,9606.ENSP00000482259 | TNFRSF1A,IL6,CCL3,CCL4 |
| GO:0043922 | Negative regulation by host of viral transcription | 2 | 15 | 2.34 | 0.0323 | 9606.ENSP00000477908,9606.ENSP00000482259 | CCL3,CCL4 |
| GO:0150078 | Positive regulation of neuroinflammatory response | 2 | 15 | 2.34 | 0.0323 | 9606.ENSP00000385675,9606.ENSP00000477908 | IL6,CCL3 |
| GO:0002682 | Regulation of immune system process | 6 | 1438 | 0.84 | 0.0324 | 9606.ENSP00000297350,9606.ENSP00000384126,9606.ENSP00000385675,9606.ENSP00000477908,9606.ENSP00000482259,9606.ENSP00000487363 | TNFRSF11B,LRRC32,IL6,CCL3,CCL4,HAVCR1 |
| GO:0006952 | Defense response | 6 | 1394 | 0.85 | 0.0324 | 9606.ENSP00000162749,9606.ENSP00000276431,9606.ENSP00000385675,9606.ENSP00000436607,9606.ENSP00000477908,9606.ENSP00000482259 | TNFRSF1A,TNFRSF10B,IL6,ADM,CCL3,CCL4 |
| GO:0009607 | Response to biotic stimulus | 6 | 1375 | 0.86 | 0.0324 | 9606.ENSP00000162749,9606.ENSP00000276431,9606.ENSP00000385675,9606.ENSP00000436607,9606.ENSP00000477908,9606.ENSP00000482259 | TNFRSF1A,TNFRSF10B,IL6,ADM,CCL3,CCL4 |
| GO:0009966 | Regulation of signal transduction | 8 | 2978 | 0.64 | 0.0324 | 9606.ENSP00000162749,9606.ENSP00000252809,9606.ENSP00000276431,9606.ENSP00000339328,9606.ENSP00000385675,9606.ENSP00000436607,9606.ENSP00000477908,9606.ENSP00000482259 | TNFRSF1A,GDF15,TNFRSF10B,PLAUR,IL6,ADM,CCL3,CCL4 |
| GO:0034112 | Positive regulation of homotypic cell-cell adhesion | 2 | 17 | 2.29 | 0.0324 | 9606.ENSP00000339328,9606.ENSP00000385675 | PLAUR,IL6 |
| GO:0042221 | Response to chemical | 9 | 4010 | 0.57 | 0.0324 | 9606.ENSP00000162749,9606.ENSP00000252809,9606.ENSP00000297350,9606.ENSP00000339328,9606.ENSP00000384126,9606.ENSP00000385675,9606.ENSP00000436607,9606.ENSP00000477908,9606.ENSP00000482259 | TNFRSF1A,GDF15,TNFRSF11B,PLAUR,LRRC32,IL6,ADM,CCL3,CCL4 |
| GO:0045779 | Negative regulation of bone resorption | 2 | 17 | 2.29 | 0.0324 | 9606.ENSP00000297350,9606.ENSP00000385675 | TNFRSF11B,IL6 |
| GO:0048245 | Eosinophil chemotaxis | 2 | 17 | 2.29 | 0.0324 | 9606.ENSP00000477908,9606.ENSP00000482259 | CCL3,CCL4 |
| GO:0050729 | Positive regulation of inflammatory response | 3 | 145 | 1.53 | 0.0324 | 9606.ENSP00000162749,9606.ENSP00000385675,9606.ENSP00000477908 | TNFRSF1A,IL6,CCL3 |
| GO:1903978 | Regulation of microglial cell activation | 2 | 18 | 2.26 | 0.0324 | 9606.ENSP00000385675,9606.ENSP00000477908 | IL6,CCL3 |
| GO:0042981 | Regulation of apoptotic process | 6 | 1462 | 0.83 | 0.0338 | 9606.ENSP00000162749,9606.ENSP00000276431,9606.ENSP00000339328,9606.ENSP00000385675,9606.ENSP00000436607,9606.ENSP00000477908 | TNFRSF1A,TNFRSF10B,PLAUR,IL6,ADM,CCL3 |
| GO:0032103 | Positive regulation of response to external stimulus | 4 | 453 | 1.16 | 0.0360 | 9606.ENSP00000162749,9606.ENSP00000385675,9606.ENSP00000477908,9606.ENSP00000482259 | TNFRSF1A,IL6,CCL3,CCL4 |
| GO:0044419 | Biological process involved in interspecies interaction between organisms | 6 | 1490 | 0.82 | 0.0360 | 9606.ENSP00000162749,9606.ENSP00000385675,9606.ENSP00000436607,9606.ENSP00000477908,9606.ENSP00000482259,9606.ENSP00000487363 | TNFRSF1A,IL6,ADM,CCL3,CCL4,HAVCR1 |
| GO:0071356 | Cellular response to tumor necrosis factor | 3 | 175 | 1.45 | 0.0413 | 9606.ENSP00000162749,9606.ENSP00000477908,9606.ENSP00000482259 | TNFRSF1A,CCL3,CCL4 |
| GO:0043410 | Positive regulation of MAPK cascade | 4 | 481 | 1.14 | 0.0414 | 9606.ENSP00000252809,9606.ENSP00000385675,9606.ENSP00000477908,9606.ENSP00000482259 | GDF15,IL6,CCL3,CCL4 |
| GO:0032101 | Regulation of response to external stimulus | 5 | 964 | 0.93 | 0.0448 | 9606.ENSP00000162749,9606.ENSP00000339328,9606.ENSP00000385675,9606.ENSP00000477908,9606.ENSP00000482259 | TNFRSF1A,PLAUR,IL6,CCL3,CCL4 |
| GO:0098542 | Defense response to other organism | 5 | 989 | 0.92 | 0.0496 | 9606.ENSP00000162749,9606.ENSP00000385675,9606.ENSP00000436607,9606.ENSP00000477908,9606.ENSP00000482259 | TNFRSF1A,IL6,ADM,CCL3,CCL4 |

Significantly associated biological processes (Gene Ontology) with accelerated NPCV progression (n=37). NPCV: percent noncalcified plaque volume progression.
